# Prevalence and Correlates of Firearm Access Among Post-9/11 US Women Veterans Using Reproductive Healthcare: a Cross-Sectional Survey

**DOI:** 10.1007/s11606-022-07587-1

**Published:** 2022-08-30

**Authors:** Lindsey L. Monteith, Ryan Holliday, Christin N. Miller, Alexandra L. Schneider, Lisa A. Brenner, Claire A. Hoffmire

**Affiliations:** 1 VA Rocky Mountain Mental Illness, Research, Education and Clinical Center (MIRECC) for Suicide Prevention, Rocky Mountain Regional VAMC, 1700 N. Wheeling Street, Aurora, CO 80045 USA; 2 Department of Psychiatry, University of Colorado Anschutz Medical Campus, Aurora, CO USA; 3 Department of Physical Medicine and Rehabilitation, University of Colorado Anschutz Medical Campus, Aurora, CO USA; 4 Department of Neurology, University of Colorado Anschutz Medical Campus, Aurora, CO USA

**Keywords:** women, Veteran, firearms, interpersonal violence, suicide

## Abstract

**Background:**

Suicide rates have increased among women Veterans, with increased use of firearms as the method. Addressing suicide risk in this population requires understanding the prevalence and correlates of firearm access in healthcare settings frequented by women Veterans.

**Objectives:**

Characterize the prevalence and correlates of firearm ownership and storage practices among women Veterans using Department of Veterans Affairs (VA) reproductive healthcare (RHC) services.

**Design:**

Cross-sectional national survey conducted in 2018–2019 (17.9% response rate).

**Participants:**

Post-9/11 women Veterans using RHC (*n*=350).

**Main Measures:**

VA Military Sexual Trauma Screen, PTSD Checklist for DSM-5, Hurt/Insult/Threaten/Scream, Columbia-Suicide Severity Rating Scale screener, self-reported firearm access.

**Key Results:**

38.0% (95% confidence interval [95% CI]: 32.9, 43.3) of participants reported personally owning firearms, and 38.9% (95% CI: 33.7, 44.2) reported other household members owned firearms. Among those with firearms in or around their homes, 17.8% (95% CI: 12.3, 24.4) and 21.9% (95% CI: 15.9, 28.9) reported all were unsafely stored (loaded or unlocked, respectively). Women who experienced recent intimate partner violence were less likely to report personally owning firearms (adjusted prevalence ratio [APR]=0.75; 95% CI: 0.57, 0.996). Those who experienced military sexual harassment (APR=1.46; 95% CI=1.09, 1.96), were married (APR=1.74; 95% CI: 1.33, 2.27), or lived with other adult(s) (APR=6.26; 95% CI: 2.87, 13.63) were more likely to report having household firearms owned by someone else. Storing firearms loaded was more prevalent among women with lifetime (APR=1.47; 95% CI=1.03, 2.08) or past-month (APR=1.69; 95% CI=1.15, 2.48) suicidal ideation and less likely among those with other adult(s) in the home (unadjusted PR=0.62; 95% CI=0.43, 0.91). Those with parenting responsibilities (APR=0.61; 95% CI=0.38, 0.97) were less likely to store firearms unlocked.

**Conclusions:**

Firearm access is prevalent among post-9/11 women Veterans using VA RHC. Interpersonal factors may be important determinants of firearm access in this population. Safe firearm storage initiatives are needed among women Veterans using RHC, particularly for those with suicidal ideation.

**Supplementary Information:**

The online version contains supplementary material available at 10.1007/s11606-022-07587-1.

## INTRODUCTION

From 2005 to 2018, female Veterans experienced an increase in their age-adjusted suicide rate (55.6%) that exceeded that of male Veterans (43.5%) and female non-Veterans (34.5%).^1^ Consequently, in 2018, the age-adjusted suicide rate was 2.1 times higher for women Veterans than women non-Veterans.^2^ Within the Veteran population, suicide rates are highest among younger Veterans (ages 18–34),^2^ many of whom served post-9/11. Thus, there is a particular need to prevent suicide among younger post-9/11 women Veterans.

One promising approach to addressing this is implementing upstream suicide prevention initiatives within settings where women Veterans frequently receive services. While such strategies are effective in reducing suicide, less is known regarding how and where to tailor these approaches. Settings providing reproductive healthcare (RHC) may be applicable given how frequently women Veterans seek RHC from the Veterans Health Administration (VHA)^3^ and considering that reproductive health conditions are top health concerns reported by women Veterans under age 45.^4^ In particular, 43% of women Veterans in VHA care have a reproductive health diagnosis.^5^ Moreover, reproductive health concerns are often comorbid with mental health diagnoses,^5^ which can exacerbate suicide risk. Finally, given the intimate nature of RHC, women often develop sustained relationships with their RHC providers built upon trust,^6^ which is paramount to suicide risk assessment and prevention.^7^

An essential component to beginning to integrate suicide prevention initiatives into VHA RHC settings is understanding lethal means access among women Veterans accessing VHA RHC. As firearms are the leading means of suicide among women Veterans,^2^ better understanding of firearm access among women Veterans using VHA RHC would ensure upstream prevention services integrated into RHC settings are tailored to the needs of women Veterans reached by such services. Addressing firearm access in RHC settings has precedence; the American College of Obstetricians and Gynecologists recommends “periodic injury prevention evaluation and counseling regarding firearms.”^8^ This recommendation stems from the fact that firearm access, which can occur through personal or household ownership or unsafe storage (e.g., unsecured, loaded), is associated with increased risk for suicide,^9–11^ as well as numerous other exposures and negative health outcomes that can and should be addressed in RHC settings (e.g., intimate partner violence, household/child firearm safety).^8^

Interpersonal violence, which is highly prevalent among women Veterans, may influence firearm access in this population. Monteith and colleagues^12^ found that military sexual trauma [MST] was a prominent theme driving firearm ownership and unsafe storage among women Veterans. Sexual assault, intimate partner violence (IPV), and post-traumatic stress disorder (PTSD) were also associated with keeping a firearm or other weapon nearby to feel safe in a larger sample of active component and Reserve/National Guard women.^13^

Interpersonal factors, such as marital status and parenting responsibilities, are relevant to the healthcare provided in RHC settings and appear salient to women Veterans’ firearm storage practices. For example, individuals with children in the home report safer storage (e.g., locked, unloaded),^12,14^ and this association is particularly strong among women.^15^ Women who are the primary caregivers of children have also reported being less likely to keep a weapon nearby to feel safe.^13^ RHC providers are well-poised to leverage the importance of safety for all family members, including children, in the home when addressing firearm safety with their patients.

Nonetheless, firearm studies have generally taken a gender-neutral approach, rarely reporting on firearm access by gender or sex. Prior studies reporting on rates of firearm access among women Veterans have yielded informative findings, but have been sparse. For example, in a nationally representative study of US adults, 24.4% of women Veterans (*n*=38) reported owning firearms and 14.4% reported residing in a household with firearm(s).^16^ Additionally, 13.2% of women Veteran firearm owners reported storing firearms both loaded and unlocked, and 45.4% reported either storing firearms loaded and locked or unloaded and unlocked.^14^ In other samples that included women Veterans, rates of household firearm access ranged from 30.7 to 39.2%.^14,17^ However, to date, no studies have examined the extent to which women Veterans using RHC own and safely store firearms, which is critical to informing upstream suicide prevention for women Veterans in RHC.

To address this, we sought to characterize the extent to which women Veterans using VHA RHC personally owned firearms, had household firearms that they did not personally own, and engaged in unsafe firearm storage practices (e.g., loaded, unlocked). We also aimed to identify factors associated with women Veterans’ personal and household firearm ownership and storage, examining interpersonal factors (marital status, adult household composition, parenting responsibilities), trauma exposure and sequelae (MST, IPV, provisional PTSD), and suicidal ideation (SI) and attempt.

## METHODS

### Participants and Procedures

This analysis was part of a larger mixed-methods study aimed at understanding suicide risk and prevention in VHA RHC settings. We used data from the Department of Veterans Affairs (VA) Corporate Data Warehouse (CDW) and VA-Department of Defense (DoD) Identity Repository to construct a random sample of 2250 post-9/11 women Veterans stratified by age and region. Inclusion criteria included being of reproductive age (18–44 years) when separating from military service and using RHC that VA provided and/or paid for in the Fiscal Year 2018. All participants separated between 10/1/2009 and 9/30/2018; thus, as some women were age 44 when separating from service in 2009, the survey sample included women with ages up to 53. RHC use was defined as having documented gynecology or women’s surgeries encounters, medical encounters outside of these settings associated with ICD-10 code(s) for qualifying reproductive health conditions or procedures and/or CPT codes for common gynecological procedures, or pharmacy fills for medications indicated solely for reproductive health conditions or contraception (more detailed information is available in Supplemental Table 1).

From 12/2018 to 6/2019, women in the identified cohort received three invitation letters, sent 4 weeks apart, to participate in a survey. To facilitate recruitment following an initial recruitment wave, a randomly selected portion also received a study flyer, paper survey, and return envelope in each mailing.^18^ Participants consented and received $20 for participating. The local Institutional Review Board approved this study.

Mailings were returned undeliverable for 129 individuals (5.7%). Of 381 who initiated the survey, 10 were ineligible, 1 opted out, and 18 did not complete the survey, resulting in 352 eligible individuals who completed the survey (response rate of 17.9%).^18^ After removing two participants (0.6%) with missing firearm data, the final sample included 350 women Veterans.

### Measures

A brief overview regarding measures is included below, with more details in Supplemental Table 2.

#### Firearm Ownership and Storage

The survey assessed current personal and household firearm ownership. Those who endorsed either were asked if firearms were stored in or around their homes. Participants who answered affirmatively were asked if firearms in or around their homes were stored loaded and locked. Response options were dichotomized to reflect having household firearms loaded (0=none; 1=some or all) or unlocked (0=none; 1=some or all). Items were based on questions administered previously,^15,19^ with minor wording modifications.

#### MST

The standard VA MST screen, used extensively within VA and which has demonstrated construct validity,^20^ assessed the most severe MST experienced (none, military sexual harassment, military sexual assault). This approach is consistent with studies finding differential health impacts based on MST severity.^21,22^

#### IPV

A Hurt/Insult/Threaten/Scream (HITS)^23^ score ≥6 was used to screen for lifetime and past 12-months IPV, which has demonstrated good sensitivity and specificity with women Veterans.^24,25^

#### PTSD

The PTSD Checklist for DSM-5 (PCL-5)^26^ was administered to determine current provisional PTSD diagnosis and has strong test-retest reliability, internal consistency, and convergent and divergent validity.^27^

#### Suicidal Ideation and Attempt

The Columbia-Suicide Severity Rating Scale (C-SSRS)^28^ self-report screener assessed past-month and lifetime SI and lifetime suicide attempt.

#### Demographics and Military Service

Additional questions assessed race, ethnicity, age, sexual orientation, branch, deployment, combat zone, pre-9/11 military service, marital status, adult household composition, and parenting responsibilities for children under age 18. Rurality was assessed based on the urban, rural, and highly rural designations attributed to the geocoded CDW address. Region was assessed based on state of mailing address.

### Analytic Plan

Analyses were conducted in SAS, v9.4, and R, v3.6.0. For our first aim, we computed frequencies with 95% confidence intervals (CIs) for our four outcomes of interest: personal firearm ownership; household firearm ownership; and, in the subsample with firearms stored in or around their homes, firearm(s) stored loaded or unlocked (excluding responses of “unsure”).

To determine covariates, chi-square or Fisher’s exact tests were used to determine if there were significant differences based on study outcomes regarding age, race, ethnicity, sexual orientation, branch, deployment, combat zone, post-Vietnam/Peacetime service, Desert Storm/Shield service, region, and rurality. Consistent with other studies on firearm access,^14,16,29–31^ the following significantly differed between groups (*p*<.05) and were included as covariates in adjusted models: age and rurality (personal ownership); rurality and sexual orientation (household ownership); age and combat zone service (unlocked firearms). No potential covariates were significant for models examining loaded firearms.

Log-binomial models were fit to examine unadjusted and adjusted associations between correlates of interest and firearm variables. For all models, we present *p*-values alongside effect estimates, in accordance with guidance by Perneger^32^ and Rothman^33^, to allow readers to judge clinical and statistical significance.

Sensitivity analyses were conducted in which household firearm ownership was included as an additional covariate in the model with personal firearm ownership as the outcome, and personal firearm ownership as an additional covariate in adjusted analyses when examining household firearms as the outcome. Furthermore, for firearm storage analyses, a sensitivity analysis was conducted with the subgroup reporting personal firearm ownership.

## RESULTS

### Participants

Table 1 includes participant characteristics. In our sample, 53.98% (95% CI: [48.61, 59.27]; *n*=190) of participants reported any firearm access (personal and/or household firearms). Specifically, 38.00% (95% CI: [32.89, 43.32]; *n*=133) reported personally owning firearm(s), and 38.85% (95% CI: [33.72, 44.18]; *n*=136) reported that someone else in their household owned firearm(s). Among those reporting firearm access, this most frequently occurred through both personal and household firearms (41.58%; *n*=79), rather than exclusively through personal (28.42%; *n*=54) or household (30.00%; *n*=57) ownership.

**Table 1:** Full Sample Descriptives (*n*=350)

**Characteristic**	***n*** **(%)**
**Age**	
18–29	97 (27.95%)
30–35	129 (37.18%)
36–53	121 (34.87%)
**Race**	
White	231 (66.19%)
Black	55 (15.76%)
Native American/Alaskan Native	7 (2.01%)
Asian/Pacific Islander	14 (4.01%)
Multi-racial	30 (8.60%)
Other	12 (3.44%)
**Ethnicity**	
Hispanic	53 (15.19%)
Non-Hispanic	296 (84.81%)
**Sexual orientation**	
Heterosexual	286 (82.42%)
^†^LGBQ + A	61 (17.58%)
**Branch of service**	
Army	163 (46.84%)
Air Force	86 (24.71%)
Navy	63 (18.10%)
Marines/Coast Guard	43 (12.29%)
**Deployment**	
None	112 (32.94%)
Single	120 (35.29%)
Multiple	108 (31.76%)
**Combat zone**	
Yes	183 (53.82%)
No	157 (46.18%)
**Post-Vietnam/Peacetime**	
Yes	6 (1.72%)
No	343 (98.28%)
**Desert Storm/Shield**	
Yes	44 (12.61%)
No	305 (87.39%)
**OEF/OIF**	
Yes	347 (99.43%)
No	2 (0.57%)
**Region**	
Northeast	38 (10.89%)
Midwest	70 (20.06%)
South	174 (49.86%)
West	67 (19.20%)
**Rurality**	
Urban	271 (77.65%)
Rural/highly rural	78 (22.35%)
**Marital status**	
Married/remarried	150 (42.86%)
Other	200 (57.14%)
**Parenting responsibilities**	
No	178 (51.59%)
Yes	167 (48.41%)
**Military sexual trauma**	
None	97 (29.94%)
Sexual harassment	78 (24.07%)
Sexual assault	149 (45.99%)
^‡^**IPV – lifetime**	
Yes	288 (82.29%)
No	62 (17.71%)
^‡^**IPV – past-year**	
Yes	134 (38.40%)
No	215 (61.60%)
^§^**Provisional PTSD**	
Yes	156 (44.70%)
No	193 (55.30%)
^‖^**SI – lifetime**	
Yes	146 (42.07%)
No	201 (57.93%)
^‖^**SI – past-month**	
Yes	38 (10.95%)
No	309 (89.05%)
^¶^**SA - lifetime**	
Yes	81 (23.28%)
No	267 (76.72%)
**Multiple adult household**	
Yes	263 (76.01%)
No	83 (23.99%)

*Note*. ^†^*LGBQ+A* lesbian, gay, bisexual, questioning + asexual; ^‡^*IPV* intimate partner violence; ^§^*PTSD* post-traumatic stress disorder; ^‖^*SI* suicidal ideation; ^¶^*SA* suicide attempt

Data missing for the following variables for personal and household firearms: age (*n*=3), race (*n* = 1), ethnicity (*n* = 1), sexual orientation (*n* = 3), branch of service (*n* = 2), deployment (*n* = 10), combat zone (*n* = 10), service era (*n* = 1), region (*n* = 1), rurality (*n* = 1), parenting responsibilities (*n* = 5), multiple adult household (n = 5), military sexual trauma (*n* = 26), IPV past-year (*n* = 1), PTSD (*n* = 1), SI lifetime (*n* = 3), SI past-month (*n* = 3), SA lifetime (*n* = 2)

Among those with firearm access, 88.95% (95% CI: [83.60, 93.03]; *n*=169) indicated firearms were stored in or around their homes. Of those, 17.75% (95% CI: [12.31, 24.36]; *n*=30) reported *all* firearms were stored loaded, 22.49% (95% CI: [16.43, 29.54]; *n*=38) reported *some* were stored loaded, 52.66% (95% CI: [44.85, 60.38]; *n*=89) reported *none* were stored loaded, and 7.10% (95% CI: [3.72, 12.08]; *n*=12) reported being unsure. Additionally, 21.89% (95% CI: [15.91, 28.89]; *n*=37) reported *all* were stored unlocked, 14.79% (95% CI: [9.81, 21.06]; *n*=25) reported *some* were stored unlocked, 59.76% (95% CI: [51.96, 67.22]; *n*=101) reported *none* were stored unlocked, and 3.55% (95% CI: [1.31, 7.57]; *n*=6) were unsure.

### Between-Group Differences (Table 2)

There were significant between-group differences in personal firearm ownership by age (*χ*^2^=10.79, *p*=.0045) and rurality (*χ*^2^=6.02, *p*=.014): those owning firearms tended to be older and live in urban settings. There were significant differences in household firearm ownership by rurality (*χ*^2^=9.35, *p*=.0022), sexual orientation (*χ*^2^=9.93, *p*=.0016), marital status (*χ*^2^=23.15, *p*<0.0001), adult household composition (*χ*^2^=46.70, *p*<0.001), parenting responsibilities (*χ*^2^=7.80, *p*=.0052), and MST (*χ*^2^= 9.41, *p*=.0090), with household firearm ownership more common among those who were in urban settings, heterosexual, married, had other adult(s) residing in the home, had parental responsibilities, and had experienced military sexual harassment.

**Table 2:** Between-Group Differences Based on Firearm Ownership and Firearm Storage Practices Among Women Veterans

	**Personal firearm ownership**		**Household firearm ownership**		**Loaded vs unloaded**		**Unlocked vs locked**	
	**Yes** (*n*=133)	**No** (*n*=217)	**Yes** (*n*=136)	**No** (*n*=214)	Loaded^*^ (*n*=68)	**Unloaded** *(n=*89)	**Unlocked**^†^ (*n*=62)	**Locked** *(n=*101)
**Age**								
18–29	**29 (21.97%)**	**68 (31.63%)**	39 (28.68%)	58 (27.49%)	18 (26.47%)	19 (21.59%)	**23 (37.70%)**	**15 (14.85%)**
30–35	**43 (32.58%)**	**86 (40.00%)**	50 (36.76%)	79 (37.44%)	21 (30.88%)	39 (44.32%)	**17 (27.87%)**	**45 (44.55%)**
36–53	**60 (45.45%)**	**61 (28.37%)**	47 (34.56%)	74 (35.07%)	29 (42.65%)	30 (34.09%)	**21 (34.43%)**	**41 (40.59%)**
**Race**								
White	94 (71.21%)	137 (63.13%)	101 (74.26%)	130 (61.03%)	48 (70.59%)	64 (72.73%)	47 (77.05%)	70 (69.31%)
Black	17 (12.88%)	38 (17.51%)	14 (10.29%)	41 (19.25%)	7 (10.29%)	12 (13.64%)	4 (6.56%)	16 (15.84%)
Native American/Alaskan Native	2 (1.52%)	5 (2.30%)	3 (2.21%)	4 (1.88%)	1 (1.47%)	2 (2.27%)	1 (1.64%)	2 (1.98%)
Asian/Pacific Islander	4 (3.03%)	10 (4.61%)	6 (4.41%)	8 (3.76%)	2 (2.94%)	3 (3.41%)	2 (3.28%)	3 (2.97%)
Multi-racial	11 (8.33%)	19(8.76%)	9 (6.62%)	21 (9.86%)	8 (11.76%)	3 (3.41%)	6 (9.84%)	6 (5.94%)
Other	4 (3.03%)	8 (3.69%)	3 (2.21%)	9 (4.23%)	2 (2.94%)	4 (4.55%)	1 (1.64%)	4 (3.96%)
**Ethnicity**								
Hispanic	19 (14.39%)	34 (15.67%)	15 (11.03%)	38 (17.84%)	7 (10.29%)	12 (13.64%)	6 (9.84%)	13 (12.87%)
Non-Hispanic	113 (85.61%)	183 (84.33%)	121 (88.97%)	175 (82.16%)	61 (89.71%)	76 (86.36%)	55 (90.16%)	88 (87.13%)
**Sexual orientation**								
Heterosexual	111 (84.73%)	175 (81.02%)	**123 (90.44%)**	**163 (77.25%)**	55 (82.09%)	77 (87.50%)	49 (81.67%)	88 (87.13%)
^§^LGBQ+A	20 (15.27%)	41 (18.98%)	**13 (9.56%)**	**48 (22.75%)**	12 (17.91%)	11 (12.50%)	11 (18.33%)	13 (12.87%)
**Branch of service**								
Army	60 (45.11%)	103 (47.91%)	57 (44.22%)	106 (46.77%)	30 (44.12%)	41 (46.07%)	23 (37.70%)	51 (50.50%)
Air Force	33 (24.81%)	53 (24.65%)	33 (24.44%)	53 (24.88%)	16 (23.53%)	24 (26.97%)	18 (29.51%)	23 (22.77%)
Navy	20 (15.04%)	43 (20.00%)	25 (18.52%)	38 (17.84%)	12 (17.65%)	14 (15.73%)	11 (18.03%)	15 (14.85%)
Marines/Coast Guard	22 (16.54%)	21 (9.68%)	21 (15.44%)	22 (10.28%)	10 (14.71%)	12 (13.48%)	10 (16.13%)	13 (12.87%)
**Deployment**								
None	35 (27.13%)	77 (36.49%)	43 (32.33%)	69 (33.33%)	19 (29.69%)	26 (29.55%)	22 (37.29%)	27 (27.00%)
Single	46 (35.66%)	74 (35.07%)	46 (34.59%)	74 (35.75%)	25 (39.06%)	29 (32.95%)	17 (28.81%)	39 (39.00%)
Multiple	48 (37.21%)	60 (28.44%)	44 (33.08%)	64 (30.92%)	20 (31.25%)	33 (37.50%)	20 (33.90%)	34 (34.00%)
**Combat zone**								
Yes	74 (57.36%)	109 (51.66%)	71 (53.38%)	112 (54.11%)	37 (57.81%)	50 (56.82%)	**27 (45.76%)**	**62 (62.00%)**
No	55 (42.64%)	102 (48.34%)	62 (46.62%)	95 (45.89%)	27 (42.19%)	38 (43.18%)	**32 (54.24%)**	**38 (38.00%)**
**Post-Vietnam/Peacetime**								
Yes	1 (0.75%)	5 (2.31%)	2 (1.47%)	4 (1.88%)	0 (0.00 %)	2 (2.25%)	0 (0.00%)	2 (1.98%)
No	132 (99.25%)	211 (97.69%)	134 (98.53%)	209 (98.12%)	68 (100.00%)	87 (97.75%)	62 (100.00%)	99 (98.02%)
**Desert Storm/Shield**								
Yes	20 (15.04%)	24 (11.11%)	20 (14.71%)	24 (11.27%)	7 (10.29%)	15 (16.85%)	8 (12.90%)	14 (13.86%)
No	113 (84.96%)	192 (88.89%)	116 (85.29%)	189 (88.73%)	61 (89.71%)	74 (83.15%)	54 (87.10%)	87 (86.14%)
**Region**								
Northeast	8 (6.02%)	30 (13.89%)	8 (5.88%)	30 (14.08%)	4 (5.88%)	5 (5.62%)	2 (3.23%)	7 (6.93%)
Midwest	27 (20.30%)	43 (19.91%)	31 (22.79%)	39 (18.31%)	14 (20.59%)	19 (21.35%)	12 (19.35%)	22 (21.78%)
South	71 (53.38%)	103 (47.69%)	67 (49.26%)	107 (50.23%)	37 (54.41%)	41 (46.07%)	36 (58.06%)	48 (47.52%)
West	27 (20.30%)	40 (18.52%)	30 (22.06%)	37 (17.37%)	13 (19.12%)	24 (26.97%)	12 (19.35%)	24 (23.76%)
**Rurality**								
Urban	**94 (70.68%)**	**177 (81.94%)**	**94 (69.12%)**	**177 (83.10%)**	47 (69.12%)	67 (75.28%)	45 (72.58%)	76 (75.25%)
Rural/highly rural	**39 (29.32%)**	**39 (18.06%)**	**42 (30.88%)**	**36 (16.90%)**	21 (30.88%)	22 (24.72%)	17 (27.42%)	25 (24.75%)
**Marital status**								
Married/remarried	64 (48.12%)	86 (39.63%)	**80 (58.82%)**	**70 (32.71%)**	35 (51.47%)	42 (47.19%)	25 (40.32%)	53 (52.48%)
Other	69 (51.88%)	131 (60.37%)	**56 (41.18%)**	**144 (67.29%)**	33 (48.53%)	47 (52.81%)	37 (59.68%)	48 (47.52%)
**Multiple adult household**								
Yes	57 (16.52%)	157 (73.36%)	**129 (95.56%)**	**133 (63.33%)**	**51 (77.27%)**	**80 (89.89%)**	47 (78.33%)	90 (89.11%)
No	26 (19.85%)	105 (80.15%)	**6 (4.44%)**	**77 (36.67%)**	**15 (22.73%)**	**9 (10.11%)**	13 (21.67%)	11 (10.89%)
**Parenting responsibilities**								
No	62 (47.33%)	116 (54.21%)	**57 (42.22%)**	**121 (57.62%)**	35 (53.03%)	35 (39.33%)	**38 (63.33%)**	**36 (35.64%)**
Yes	69 (52.67%)	98 (45.79%)	**78 (57.78%)**	**89 (42.38%)**	31 (46.97%)	54 (60.67%)	**22 (36.67%)**	**65 (64.36%)**
**Military sexual trauma**								
None	32 (25.60%)	65 (32.66%)	**38 (29.46%)**	**59 (30.26%)**	15 (23.81%)	26 (30.59%)	17 (28.81%)	24 (25.26%)
Sexual Harassment	34 (27.20%)	44 (22.11%)	**42 (32.56%)**	**36 (18.46%)**	22 (34.92%)	22 (25.88%)	21 (35.59%)	27 (28.42%)
Sexual Assault	59 (47.20%)	90 (45.23%)	**49 (37.98%)**	**100 (51.28%)**	26 (41.27%)	37 (43.53%)	21 (35.59%)	44 (46.32%)
^‖^**IPV – lifetime**								
Yes	108 (81.20%)	180 (82.95%)	115 (84.56%)	173 (80.84%)	57 (83.82%)	73 (82.02%)	52 (83.87%)	84 (83.17%)
No	25 (18.80%)	37 (17.05%)	21 (15.44%)	41 (19.16%)	11 (16.18%)	16 (17.98%)	10 (16.13%)	17 (16.83%)
^‖^**IPV – past-year**								
Yes	45 (33.83%)	89 (41.20%)	50 (36.76%)	84 (39.44%)	26 (38.24%)	33 (37.08%)	24 (38.71%)	34 (33.66%)
No	88 (66.17%)	127 (58.80%)	86 (63.24%)	129 (60.56%)	42 (61.76%)	56 (62.92%)	38 (61.29%)	67 (66.34%)
**Provisional** ^¶^**PTSD**								
Yes	57 (42.86%)	99 (45.83%)	57 (41.91%)	99 (46.48%)	30 (44.12%)	32 (35.96%)	20 (32.26%)	43 (42.57%)
No	76 (57.14%)	117 (54.17%)	79 (58.09%)	114 (53.52%)	38 (55.88%)	57 (64.04%)	42 (67.74%)	58 (57.43%)
^#^**SI – lifetime**								
Yes	50 (37.88%)	96 (44.65%)	56 (41.18%)	90 (42.65%)	**33 (48.53%)**	**28 (31.82%)**	27 (43.55%)	38 (38.00%)
No	82 (62.12%)	119 (55.35%)	80 (58.82%)	121 (57.35%)	**35 (51.47%)**	**60 (68.18%)**	35 (56.45%)	62 (62.00%)
^#^**SI – past-month**								
Yes	11 (8.33%)	27 (12.56%)	12 (8.82%)	26 (12.32%)	**11 (16.18%)**	**5 (5.68%)**	5 (8.06%)	11 (11.00%)
No	121 (91.67%)	188 (87.44%)	124 (91.18%)	185 (87.68%)	**57 (83.82%)**	**83 (94.32%)**	57 (91.94%)	89 (89.00%)
^**^SA - lifetime								
Yes	25 (18.80%)	56 (26.05%)	26 (19.12%)	55 (25.94%)	15 (22.06%)	16 (17.98%)	13 (20.97%)	19 (18.81%)
No	108 (81.20%)	159 (73.95%)	110 (80.88%)	157 (74.06%)	53 (77.94%)	73 (82.02%)	49 (79.03%)	82 (81.19%)

*Note*. This table displays frequencies and percentages. Significant *p*-values (<.05) from chi-square tests and Fisher’s exact tests (for race and post-Vietnam/Peacetime service) are bolded

^*^Some or all firearms are stored loaded; ^†^some or all firearms are stored unlocked; ^§^*LGBQ+A* lesbian, gay, bisexual, questioning + asexual; ^‖^*IPV* intimate partner violence; ^¶^*PTSD* post-traumatic stress disorder; ^#^*SI* suicidal ideation; ^**^SA suicide attempt

Data missing for the following variables for firearm ownership: age (*n*=3), race (*n* = 1), ethnicity (*n* = 1), sexual orientation (*n* = 3), branch of service (*n* = 2 for personal); deployment (*n* = 10), combat zone (*n* = 10), era (*n* = 1), region (*n* = 1), rurality (*n* = 1), parenting responsibilities (*n* = 5), multiple adult household (*n* = 5), military sexual trauma (*n* = 26), IPV – past-year (*n* = 1), PTSD (*n* = 1), SI lifetime (*n* = 3), SI past-month (*n* = 3), SA lifetime (*n* = 2)

Data missing for the following variables for firearm storage practices: age (*n*=1), race (*n* = 1), ethnicity (*n* = 1), sexual orientation (*n* = 2), parenting responsibilities (*n* = 2), branch of service (*n* = 1); deployment (*n* = 5), combat zone (*n* = 5), military sexual trauma (*n* = 11), SI lifetime (*n* = 1), SI past-month (*n* = 1)

Significant between-group differences occurred in storing firearms loaded based on adult household composition (*χ*^2^=4.61, *p*=.032) lifetime SI (*χ*^2^=4.50, *p*=.034), and past-month SI (*χ*^2^=4.60, *p*=.032); those living in a household with other adult(s) were less likely to store firearms loaded, whereas those experiencing SI were more likely to store firearms loaded. For storing firearms unlocked, there were significant differences in age (*χ*^2^=11.61, *p*=.0030), combat zone service (*χ*^2^=3.97, *p*=.046), and parenting responsibilities (*χ*^2^=11.62, *p*=.0007); those younger, without combat zone service, and without parenting responsibilities were more likely to report storing firearms unlocked.

### Personal Firearms (Table 3)

Adjusting for age and rurality, women who experienced recent IPV were 24.86% less likely to report personal firearm ownership: adjusted PR (APR)=0.75 [95% CI=0.57, 0.996]. In the sensitivity analysis adjusting for household firearms, recent IPV was no longer associated with personal ownership: APR=0.83 [95% CI=0.65, 1.07].

**Table 3:** Factors Associated with Personal Firearm Ownership Among Women Veterans

	**Unadjusted**			Adjusted*		
**Variable**	***n***	**Prevalence ratio [95% confidence interval]**	***p***	***n***	**Prevalence ratio [95% confidence interval]**	***p***
Military sexual trauma (MSH^†^ only vs none)	324	1.32 [0.90, 1.93]	0.15	321	1.26 [0.87, 1.83]	0.22
Military sexual trauma (MSA^‡^ vs none)	324	1.20 [0.85, 1.70]	0.30	321	1.24 [0.88, 1.74]	0.21
Intimate partner violence - lifetime	350	0.93 [0.66, 1.30]	0.67	346	0.88 [0.63, 1.23]	0.46
**Intimate partner violence - recent**	349	0.82 [0.62, 1.09]	0.18	345	**0.75 [0.57, 0.996]**	**0.047**
Provisional ^§^PTSD	349	0.93 [0.71, 1.22]	0.59	345	0.94 [0.73, 1.22]	0.65
Marital status	350	1.24 [0.95, 1.61]	0.12	346	1.12 [0.86, 1.45]	0.40
Multiple adult household	345	1.28 [0.90, 1.82]	0.17	341	1.17 [0.83, 1.66]	0.36
Parenting responsibilities	345	1.19 [0.91, 1.55]	0.22	341	1.14 [0.87, 1.49]	0.33
Suicidal ideation - lifetime	347	0.84 [0.63, 1.11]	0.22	343	0.88 [0.67, 1.15]	0.34
Suicidal ideation - past-month	347	0.74 [0.44, 1.24]	0.25	343	0.75 [0.45, 1.24]	0.26
Suicide attempt - lifetime	348	0.76 [0.53, 1.09]	0.14	344	0.81 [0.57, 1.15]	0.23

*Note*. *n*s refer to sample sizes across analyses, which vary due to specific variables having some missing data

^*^Adjusted for age and rurality

^†^*MSH* military sexual harassment

^‡^*MSA* military sexual assault

^§^*PTSD* post-traumatic stress disorder

### Household Firearms (Table 4)

In both unadjusted and adjusted analyses, being married (APR=1.74 [95% CI=1.33, 2.27]) and having other adult(s) residing in the home (APR=6.26 [95% CI=2.87, 13.63]) were associated with increased prevalence of another household member owning firearms. Those with parenting responsibilities were also more likely to report household firearm ownership: PR=1.46 [95% CI=1.12, 1.91], but this was not significant when adjusting for rurality and sexual orientation. Although military sexual harassment was not associated with household firearms in unadjusted analyses, women who experienced military sexual harassment were more likely to report having household firearms after accounting for rurality and sexual orientation: APR=1.46 [95% CI=1.09, 1.96]. Results were similar in sensitivity analyses adjusting for personal firearm ownership.

**Table 4:** Factors Associated with Household Firearm Ownership Among Women Veterans

**Unadjusted**				Adjusted*		
**Variable**	***n***	**Prevalence ratio [95% confidence interval]**	***p***	***n***	**Prevalence ratio [95% confidence interval]**	***p***
**Military sexual trauma (MSH**^†^ **only vs none)**	324	1.37 [1.00, 1.90]	0.053	321	**1.46 [1.09, 1.96]**	**0.012**
Military sexual trauma (MSA^‡^ vs none)	324	0.84 [0.60, 1.18]	0.31	321	0.90 [0.65, 1.25]	0.53
Intimate partner violence - lifetime	350	1.18 [0.81, 1.72]	0.39	346	1.12 [0.78, 1.61]	0.55
Intimate partner violence - recent	349	0.93 [0.71, 1.23]	0.62	346	0.95 [0.74, 1.23]	0.71
Provisional ^§^PTSD	349	0.89 [0.68, 1.17]	0.41	345	0.84 [0.65, 1.08]	0.18
**Marital status**	350	**1.90 [1.46, 2.49]**	**<0.001**	346	**1.74 [1.33, 2.27]**	**<0.001**
**Multiple adult household**	345	**6.81 [3.12, 14.86]**	**<0.001**	341	**6.26 [2.87, 13.63]**	**<0.001**
**Parenting responsibilities**	345	**1.46 [1.12, 1.91]**	**0.006**	341	1.30 [0.99, 1.7]	0.056
Suicidal ideation - lifetime	347	0.96 [0.74, 1.26]	0.79	343	1.04 [0.81, 1.34]	0.77
Suicidal ideation - past-month	347	0.79 [0.48, 1.28]	0.34	343	0.78 [0.49, 1.25]	0.31
Suicide attempt - lifetime	348	0.78 [0.55, 1.1]	0.16	344	0.83 [0.6, 1.17]	0.29

*Note*. Significant *p*-values (<.05) are bolded. *n*s refer to sample sizes across analyses, which vary due to there being some missing data for specific variables

^*^Adjusted for rurality and sexual orientation

^†^*MSH* military sexual harassment

^‡^*MSA* military sexual assault

^§^*PTSD* post-traumatic stress disorder

### Firearm Storage (Table 5)

#### Loaded

Participants with lifetime SI, PR=1.47 [95% CI=1.03, 2.08], or past-month SI, PR=1.69 [95% CI=1.15, 2.48], were more likely to report storing firearms loaded. These associations were not significant in the sensitivity analysis limited to personal firearm owners. Those with other adult(s) living in the home were less likely to store firearms loaded (PR=0.62 [95% CI=0.43, 0.91]).

**Table 5:** Factors Associated with Storing Firearms Loaded or Unlocked Among Women Veterans

	**Loaded**			**Unlocked**					
	**Unadjusted**			**Unadjusted**			Adjusted*		
**Variable**	***n***	**Prevalence ratio** **[95% CI**^†^**]**	***p***	***n***	**Prevalence ratio** **[95% CI**^†^**]**	***p***	***n***	**Prevalence ratio** **[95% CI**^†^**]**	***p***
Military sexual trauma (MSH^‡^ only vs none)	148	1.37 [0.83, 2.25]	0.22	154	1.06 [0.65, 1.71]	0.83	151	1.07 [0.67, 1.71]	0.78
Military sexual trauma (MSA^§^ vs none)	148	1.13 [0.68, 1.86]	0.64	154	0.78 [0.47, 1.29]	0.33	151	0.83 [0.5, 1.35]	0.45
Intimate partner violence - lifetime	157	1.08 [0.66, 1.77]	0.77	163	1.03 [0.60, 1.76]	0.91	158	1.17 [0.68, 2.01]	0.56
Intimate partner violence - recent	157	1.03 [0.71, 1.48]	0.88	163	1.14 [0.77, 1.7]	0.51	158	1.29 [0.87, 1.91]	0.20
Provisional ^‖^PTSD	157	1.21 [0.85, 1.73]	0.29	163	0.76 [0.49, 1.16]	0.20	158	0.85 [0.55, 1.32]	0.47
Marital status	157	1.10 [0.77, 1.58]	0.60	163	0.74 [0.49, 1.1]	0.14	158	0.89 [0.59, 1.35]	0.59
**Multiple adult household**	155	**0.62 [0.43, 0.91]**	**0.01**	161	**0.63 [0.41, 0.98]**	**0.04**	158	0.73 [0.48, 1.12]	0.15
**Parenting responsibilities**	155	0.73 [0.51, 1.05]	0.09	161	**0.49 [0.32, 0.75]**	**0.001**	158	**0.61 [0.38, 0.97]**	**0.04**
**Suicidal ideation - lifetime**	156	**1.47 [1.03, 2.08]**	**0.032**	162	1.15 [0.78, 1.7]	0.48	157	1.11 [0.75, 1.63]	0.60
**Suicidal ideation - past-month**	156	**1.69 [1.15, 2.48]**	**0.0078**	162	0.80 [0.38, 1.7]	0.56	157	0.73 [0.32, 1.69]	0.46
Suicide attempt - lifetime	157	1.15 [0.76, 1.75]	0.51	163	1.09 [0.68, 1.74]	0.73	158	1.11 [0.70, 1.75]	0.66

*Note*. Analyses were specific to those who reported having firearms stored in or around their homes. Significant *p*-values (<.05) are bolded. *n*s refer to sample sizes across analyses, which vary due to there being some missing data for specific variables

^*^Adjusted for age and having served in a combat zone

^†^*CI* confidence interval

^‡^*MSH* military sexual harassment

^§^*MSA* military sexual assault

^‖^*PTSD* post-traumatic stress disorder

#### Unlocked

Participants with parenting responsibilities were less likely to report storing firearms unlocked in unadjusted and adjusted analyses (APR=0.61 [95% CI=0.38, 0.97]). Results were similar in the sensitivity analysis limited to those who personally owned firearms. Those with other adult(s) living in the home were less likely to report storing firearms unlocked, but only in unadjusted analyses (PR=0.63 [95% CI=0.41, 0.98]).

## DISCUSSION

To our knowledge, this study is the first to examine the prevalence and correlates of firearm access among women Veterans using VHA RHC. Findings underscore the high prevalence of firearm access in this population, which most commonly entailed personally owning firearms *and* other household member(s) owning firearm(s). Considering the high prevalence of personal and household firearms in our sample, relative to other samples of women Veterans,^16^ it is important that providers ask about access to both personal and household firearms when assessing suicide risk with women Veterans receiving RHC. Additionally, the prevalence of unsafe firearm storage in our sample underscores the need to determine optimal ways to increase safe firearm storage (e.g., locked, unloaded) among women Veterans in RHC.

Women Veterans who were married or residing with other adult(s) were more likely to report living in households with firearms that other household members owned. However, those with other adult(s) living in the home were less likely to report storing firearms loaded or unlocked (unadjusted analyses). Thus, including other household members in lethal means safety efforts may bolster the prevention of suicide among women Veterans.

Parenting responsibilities also related to firearm storage. Women Veterans with parenting responsibilities were less likely to report storing firearms unlocked, consistent with other research.^14^ One possible explanation is that women Veterans with parenting responsibilities lock firearms to protect their children from firearm injuries. An important point of intervention may entail discussing risks posed to self and youths of storing household firearms unsafely.^34,35^ In other studies, adults with children at home were more likely to report believing it is at least sometimes appropriate for providers to discuss firearms with their patients;^36^ if supported in future research with women Veterans, this would bode well for RHC providers having such conversations with women Veterans.

Interpersonal violence was another salient factor associated with firearm behaviors. Women Veterans who experienced military sexual harassment were more likely to report having household firearms owned by others. One potential explanation is that, for women Veterans sexually harassed during military service, firearm access through another household member increases perceived safety.^37^ However, unexpectedly, neither MST nor probable PTSD was associated with personal firearm ownership or storage. Thus, specific trauma characteristics (e.g., traumatization frequency, perpetrator identity) or sequelae (e.g., PTSD hyperarousal)^38^ may be more influential.

Notably, recent IPV was associated with a *lower* likelihood of personal firearm ownership before adjusting for household firearms. This is counter to a prior study in which women who experienced lifetime IPV (threats or physical violence) were twice as likely to report keeping a weapon nearby to feel safe.^13^ One potential explanation for our finding is that women who recently experienced IPV feel unsafe owning firearms given the ongoing threat that such firearms could be used against them.^39^ Additional research is warranted to further elucidate the role of IPV in women Veterans’ firearm ownership.

Women with past-month or lifetime SI were more likely to report having loaded firearms. This finding is disconcerting as both SI and unsafe storage are risk factors for suicide.^40^ This suggests that, despite efforts to bolster safe firearm storage among Veterans,^41^ enhanced initiatives are necessary within VHA RHC settings. Recent articles have noted the import of accounting for the function of firearm access during lethal means safety discussions^12,13^ and collaboratively identifying methods to enhance safety.^42^ Nonetheless, as the association between SI and loaded firearms was not significant in the sensitivity analysis, additional research is warranted.

### Limitations

Despite minimal non-response bias^18^, the overall response rate was low, with low base rates for many constructs (e.g., recent SI, suicide attempt). The focus on younger women Veterans accessing VHA RHC also limits generalizability. Our analysis of IPV, probable PTSD, and MST as categorical variables precludes examining if the severity of these factors relates to firearm access. Additionally, although we presented *p*-values alongside effect estimates to allow readers to judge clinical and statistical significance,^32,33^ multiple comparisons can inflate Type I error. The cross-sectional design precludes drawing conclusions regarding the directionality of observed associations. Finally, the firearm items have not been psychometrically validated.

## CONCLUSIONS

This study provides knowledge regarding firearm access among women Veterans using VHA RHC. As a substantial portion of women Veterans reported personal and/or household firearms and unsafe storage, this suggests a need to assess for firearm access and storage in this population, particularly when suicide risk is elevated. Interpersonal factors (marital status, parenting responsibilities, presence of other household adults), trauma (IPV, military sexual harassment), and SI appear relevant to women Veterans’ firearm access. Incorporating these findings into suicide prevention initiatives (e.g., lethal means safety counseling) within VHA RHC settings is essential. In doing so, suicide prevention efforts can be tailored and delivered within a healthcare setting commonly accessed by women Veterans.

## Supplementary information

ESM 1 (DOCX 18 kb)

ESM 2 (DOCX 19 kb)
