# Supplementary material for: Prevalence and Correlates of Firearm Access Among Post-9/11 US Women Veterans Using Reproductive Healthcare: a Cross-Sectional Survey
Source: J Gen Intern Med. 2022 Aug 30;37(Suppl 3):714–23. doi: 10.1007/s11606-022-07587-1 (PMC9481791; doi:10.1007/s11606-022-07587-1)
Supplement: Supplementary file 1 — (DOCX 18 kb) [file 11606_2022_7587_MOESM1_ESM.docx]

**Supplemental Table 1.**

*Reproductive Health Care Use Definition for the Current Study*

| **VA RHC User Cohort Definition** | **Diagnoses (ICD-10)** | **Outpatient Procedures (CPT)** | **Inpatient Procedures (ICD-10-PCS)** | **Medication Fills** | **Encounter Type** |
| --- | --- | --- | --- | --- | --- |
| **Conditions** | | | | | |
| Menstrual disorders and endometriosis | X |  |  | X |  |
| Abnormal cervical screening | X |  |  |  |  |
| Sexually Transmitted Infections | X |  |  | X |  |
| Vaginitis | X |  |  | X |  |
| Female Infertility | X | o | o | X |  |
| Benign breast conditions | X |  |  |  |  |
| Sexual dysfunction | X |  |  | X |  |
| Urinary conditions | X |  |  |  |  |
| Other female reproductive organ conditions | X | o | o | X |  |
| Menopausal disorders & HRT | X |  |  | X |  |
| Osteoporosis | X |  |  |  |  |
| **Cancer** | | | | | |
| Gynecologic | X |  |  | X |  |
| Breast | X |  |  | X |  |
| **Pregnancy** | | | | | |
| Pregnancy test |  | o | o |  |  |
| Screening and monitoring procedures |  | o | o |  |  |
| Normal pregnancy and delivery | X | o | o | X |  |
| Prolonged pregnancy | X |  |  |  |  |
| Ectopic & molar | X |  |  |  |  |
| Miscarriage and abortion | X | o | o |  |  |
| Obstetrical complications | X |  |  | X |  |
| Pre-existing conditions complicating pregnancy | X |  |  |  |  |
| **Preventive Care** | | | | | |
| Contraception |  | X | X | X |  |
| Screening and Diagnostic procedures |  | X | X |  |  |
| **Medical Encounters** | | | | | |
| Gynecology |  |  | o |  | X |
| Women’s surgery^1^ |  |  |  |  | X |
| **Footnotes** | | | | | |
| X=used to qualify patient as a VA RHC user; o=code available but not used for the cohort definition.  ^1^While some conditions, cancers, and pregnancy may include surgery-based treatments that are not captured with the women’s surgery VA encounter type code, we did not include information from surgery procedure codes as most of those individuals would also be captured with diagnostic codes preceding the surgery. | | | | | |
