# Supplementary material for: Prevalence and Correlates of Firearm Access Among Post-9/11 US Women Veterans Using Reproductive Healthcare: a Cross-Sectional Survey
Source: J Gen Intern Med. 2022 Aug 30;37(Suppl 3):714–23. doi: 10.1007/s11606-022-07587-1 (PMC9481791; doi:10.1007/s11606-022-07587-1)
Supplement: Supplementary file 2 — (DOCX 19 kb) [file 11606_2022_7587_MOESM2_ESM.docx]

**Supplemental Table 2.**

*Measures Analyzed in the Present Study*

| **Construct** | **Measure or Question(s)** | **Timeframe** | **Notes** |
| --- | --- | --- | --- |
| **Personal firearm ownership** | *Do you own a gun (or multiple guns)?* Response options: yes; no | Currently | N/A |
| **Household firearm ownership** | *Does anyone else you live with currently own any type of gun?* Response options: yes; no | Currently | N/A |
| **Firearms stored in or around one’s home** | *If you or someone in your household owns a gun, are any guns stored in or around your home (including in a garage, outdoor storage area, or motor vehicle)?* Response options: yes; no; N/A | Currently | Asked of those who endorsed personal and/or household firearm ownership. |
| **Firearms stored loaded** | *Are guns in or around your home currently stored loaded?* Response options: Yes, all of them; Yes, some of them; None of them; I’m not sure | Currently | Asked of participants who indicated they had firearms stored in or around their homes. Responses were dichotomized to reflect household firearms stored loaded (0=none; 1=some or all). |
| **Firearms stored locked** | *Are guns in or around your home currently stored locked? (Examples include using a locked cabinet, locked box, trigger lock or cable gun lock)* Response options: Yes, all of them; Yes, some of them; None of them; I’m not sure | Currently | Asked of participants who indicated they had firearms stored in or around their homes. Responses were dichotomized to reflect household firearms stored unlocked (0=none; 1=some or all). |
| **Military sexual trauma history** | VA Military Sexual Trauma Screen:  (1) *When you were in the military, did you ever receive unwanted, threatening, or repeated sexual attention (for example, touching, cornering, pressure for sexual favors, or inappropriate verbal remarks, etc.)?* [military sexual harassment]; (2) *When you were in the military, did you have sexual contact against your will or when you were unable to say no (for example, after being forced or threatened or to avoid other consequences)?* [military sexual assault] Response options: Yes; No; Decline to respond | During military service | Coded to determine the most severe type of military sexual trauma experienced (i.e., military sexual assault, military sexual harassment, or neither). Responses of “decline to respond” were coded as missing. |
| **Intimate partner violence (lifetime, past 12 months)** | Hurt/Insult/Threaten/Scream (HITS): Participants were asked to think about “any romantic relationships you have been in” (lifetime IPV) as well as their  “current or most recent romantic relationship, in the past 12 months” (current IPV), then they rated the frequency in which they experienced physical violence, verbal aggression, threat of harm, and were screamed or cursed at within these timeframes. | Lifetime, past 12 months | The HITS includes four items, each rated on a scale of 1 to 5, for each timeframe. Items are summed, and a score ≥6 was considered a positive screen for IPV, which is consistent with prior studies. |
| **Provisional PTSD diagnosis** | PTSD Checklist for DSM-5 (PCL-5) | Past month | This measure includes 20 items, each scored on a scale ranging from 0 to 4. Individuals were considered to have a provisional PTSD diagnosis if they had at least 1 B item endorsed, 1 C item, 2 D items, and 2 E items. Endorsement of each item was operationalized as responses of ≥2. These criteria are in line with the diagnostic criteria for DSM-5 regarding symptoms. Responses were not anchored to any specific Criterion A traumatic event. |
| **Lifetime suicidal ideation** | Columbia-Suicide Severity Rating Scale (C-SSRS) self-report screener: *Have you ever actually had any thoughts of killing yourself?* Response options: Yes; No. | Lifetime |  |
| **Past-month suicidal ideation** | Columbia-Suicide Severity Rating Scale (C-SSRS) self-report screener: *Have you felt this way in the past month?* Response options: Yes; No. | Past month | Asked of those who indicated experiencing lifetime suicidal ideation as an immediate follow up question following the query about lifetime suicidal ideation. |
| **Lifetime suicide attempt** | Columbia-Suicide Severity Rating Scale (C-SSRS) self-report screener: *Have you ever done anything to harm yourself with at least some intent to end your life?* Response options: Yes; No. | Lifetime | Asked of all participants. |
| **Marital status** | *What is your current marital status?* Response options: never married; married – first and only marriage; remarried – second or later marriage; separated; divorced; widowed. | Currently | Recategorized as married/remarried vs. other (i.e., never married, separated, divorced, or widowed) |
| **Adult household composition** | *Including yourself, how many people live in your household? (Please include each person who lives and sleeps in your household most of the time.)* (Numerical response options; participants were instructed to skip the next question “if just you (1)”) *How many of these people are children under age 18?* | Currently | Analyzed as single (only the survey respondent residing in the home) vs. multiple (2+ adults living in the home) |
| **Parenting responsibilities for children under age 18** | *For how many children do you have parenting responsibilities, regardless of the child’s age?* (Numerical response options) | Currently | Recategorized as none (0) vs any (responses of ≥1) |

*Note*. Measure names are underlined. Question content is italicized.
